# Root litter quality drives the dynamic of native mineral-associated organic carbon in a temperate agricultural soil

**DOI:** 10.1007/s11104-023-06127-y

**Published:** 2023-06-21

**Authors:** Christopher Poeplau, Neha Begill, Zhi Liang, Marcus Schiedung

**Affiliations:** 1 Thünen Institute of Climate-Smart Agriculture, Bundesallee 68, 38116 Braunschweig, Germany; 2 Department of Agroecology, Aarhus University, Blichers Allé 20, Tjele, 8830 Denmark; 3 Department of Geography, University of Zurich, Winterthurerstrasse 190, Zurich, 8057 Switzerland; 4 Department of Environmental Systems Science, ETH Zurich, Universitätstrasse 16, Zurich, 8092 Switzerland

**Keywords:** C:N ratio, Soil organic carbon, Incubation, Priming, Nitrogen mining, MAOM

## Abstract

**Background and aims:**

Understanding the fate and residence time of organic matter added to soils, and its effect on native soil organic carbon (SOC) mineralisation is key for developing efficient SOC sequestration strategies. Here, the effect of litter quality, particularly the carbon-to-nitrogen (C:N) ratio, on the dynamics of particulate (POC) and mineral-associated organic carbon (MAOC) were studied.

**Methods:**

In a two-year incubation experiment, root litter samples of the C4-grass *Miscanthus* with four different C:N ratios ranging from 50 to 124 were added to a loamy agricultural topsoil. In an additional treatment, ammonium nitrate was added to the C:N 124 litter to match the C:N 50 litter input ratio. Soils were size-fractionated after 6, 12 and 24 months and δ^13^C was measured to determine the proportion of new and native POC and MAOC. Litter quality was further assessed by mid-infrared spectroscopy and compound peak analysis.

**Results:**

Litter quality strongly affected SOC dynamics, with total SOC losses of 42.5 ± 3.0% in the C:N 50 treatment and 48.9 ± 3.0% in the C:N 124 treatment after 24 months. Largest treatment effects occurred in mineralisation of native MAOC, which was strongly primed by litter addition. The N amendment in the C:N 124 treatment did not alleviate this potential N mining flux.

**Conclusion:**

Litter quality plays a major role in overall SOC dynamics, and priming for N mining from the MAOC pool could be a dominant mechanism. However, adding N did not compensate for poor litter quality, highlighting the role of litter quality beyond stoichiometric imbalances.

## Introduction

Soil organic matter plays a crucial role for soil fertility and health and represents the largest terrestrial carbon (C) pool. Increasing the global soil organic carbon (SOC) pool, especially in agricultural soils, is acknowledged to have a certain potential to reduce atmospheric carbon dioxide concentration and help mitigate climate change (Minasny et al. 2017). In order to develop strategies to efficiently increase SOC stocks and for accurate prediction of the fate of organic matter (OM) reaching the soil, drivers and processes involved in its decomposition and stabilisation need to be understood in more detail (Castellano et al. 2015; Cotrufo et al. 2015).

The longer SOC is retained in the soil, the more it contributes to climate change mitigation. Historically, the chemical stability, i.e. recalcitrance, of organic matter input was believed to be the major driver of its stability in the soil. Researchers in the second half of the last century have developed a more differentiated view with a combination of factors including substrate quality, abiotic environment and decomposer community being responsible for the biogeochemical stability of organic matter in soils (Swift et al. 1979). Research suggested that the intrinsic chemical stability of plant derived C compounds might not necessarily imply biogeochemical stability in the soil (Dungait et al. 2012; Schmidt et al. 2011) and that the recalcitrance of organic matter can determine its decomposability in the timescale of years, but not for its centennial scale stabilisation in the soil (Lützow et al. 2006; Schmidt et al. 2011). Simply spoken: a piece of lignified wood certainly resists initial decomposition longer than a fresh blade of grass, but this does not necessarily influence its mean residence time in the soil, which is rather driven by the proportion of C that enters a more stabilised SOC pool and becomes inaccessible to decomposers. Accessibility of organic matter, and thus physical stabilisation of C, have been identified as more important stabilisation mechanisms than chemical recalcitrance (Dungait et al. 2012; Nicolardot et al. 2001; Schmidt et al. 2011; Six et al. 2002). That being said, the role of litter quality, and potentially its molecular diversity (Lehmann et al. 2020), on the fate of OM in the soil and its role in SOC build-up is certainly not negligible (Kirchmann et al. 2004; Wuest and Gollany 2013). For example, in the Ultuna frame-trial, a long-term experiment in Uppsala, Sweden, different organic amendments of the same quantity, but different quality, were added to the soil every two years (Andrén and Kätterer 1997). After roughly 60 years, which is certainly beyond ‘short-term’ in SOC dynamics, very different SOC contents were established in the plots of the different treatments (Kätterer et al. 2011). The highest SOC contents were found in the plots that received peat material (3.2%), followed by sewage sludge (2.7%), farmyard manure (2.2%), saw dust (1.9%), green manure (1.5%) and straw (1.5%), while the control without external C inputs had a SOC content of 1.1%. Calibrating input type-specific partition coefficients for SOC turnover has been the focus of modelling efforts to cope with such differences in organic matter quality, while acknowledging that important mechanisms might not be accounted for in the most widespread models to accurately predict the retention of C that reaches soils from different sources (Peltre et al. 2012; Wieder et al. 2014).

In recent years, two major pathways of stabilised soil organic matter formation during the decomposition of litter or organic amendments have been highlighted (Cotrufo et al. 2015; Liang et al. 2017; Sokol et al. 2019). The first pathway is the so-called ‘entombing effect’, which refers to stabilisation of organic matter in form of microbial necromass and metabolites which are bound to mineral surfaces or in micro-aggregates. In this scenario, plant-derived C has been broken down and utilised for microbial biosynthesis and is thus also referred to as the ‘*in-vivo’* pathway of stabilisation (Bradford et al. 2013; Liang et al. 2017). The second pathway, by contrast, is called the ‘ex-vivo’ modification (Liang et al. 2017), and suggests that once extracellular enzymes have reduced the size of OM particles via transformation and decomposition, the OM resists complete decomposition due to its altered chemical composition, spatial inaccessibility and/or energetic barriers. (Barré et al. 2016; Cotrufo et al. 2015; Liang et al. 2017). Indeed, it is estimated that stabilised, i.e. mineral-associated organic carbon (MAOC) can consist of microbial and plant-derived compounds to approximately equal shares, depending on land use (Liang et al. 2019; Angst et al. 2021). It can thus be assumed, that both major pathways of stabilisation are equally important for SOC build-up.

Interestingly, it is likely that both pathways are fostered by very contrasting properties, which complicates the inclusion of even simple indicators of substrate quality, such as the carbon-to-nitrogen (C:N) ratio, in SOC turnover models. On the one hand, it is acknowledged that SOC accrual needs N, among others due to metabolic requirements of microbes. The higher the availability of N relative to C in soils, the more C can be used for anabolism and thus the *in-vivo* pathway of C stabilisation (Manzoni et al. 2017). It has even been found that soil N limitation can indeed increase total decomposition as well as native SOC mineralisation, a mechanism referred to as N mining (Craine et al. 2007; Murphy et al. 2015; Spohn and Chodak 2015). On the other hand, substrates with a high C:N ratio are acknowledged to decay much slower than substrates with a low C:N ratio. For example Taylor et al. (1989) incubated different litter materials and linked various substrate quality indicators, including lignin content and lignin/C:N ratio, to their decay rates. In that study, the C:N ratio was by far the best explanatory variable, with litter mass loss and C:N ratio being negatively correlated. In the aforementioned Ultuna experiment, the added peat material had the strongest effect on SOC build-up with an intermediate C:N ratio of 50, while sewage sludge with the second highest SOC build-up had the lowest C:N ratio of 9 (Kätterer, pers. comm.). The substrate C:N ratio could thus be either ancillary, or affect both pathways with opposing consequences for SOC build-up. This is not well resolved and requires controlled experiments at a long enough timescale to elucidate differences. Experiments looking at the effects of litter C:N ratio on litter decay (Bonanomi et al. 2013; Taylor et al. 1989) are far more common than studies trying to understand the fate of litter-derived C in the soil by following its stabilisation in the MAOC fraction (Córdova et al. 2018) or those addressing both mechanisms (Cotrufo et al. 2015) as well as potential priming effects at the same time.

Despite the notion that soil organic matter formation has a high N cost (van Groenigen et al. 2017), bioenergy crops like *Miscanthus*, with a very low N demand and rather poor litter quality, have been found to increase SOC stocks when planted on croplands (Dondini et al. 2009). In this study we aimed at elucidating the effect of particularly wide substrate C:N ratios ranging from 50 to 124 on the decomposition and stabilisation of *Miscanthus* root litter over two years in an agricultural topsoil. By adding a treatment with a high C:N ratio litter (124) and mineral N addition combined, we aimed to test if such effects would be related to N availability per se. SOC size-fractionation was used to investigate the fate of root litter into different functional C pools. The isotopic differences in δ^13^C of Miscanthus root litter (C_4_-plant) and native SOC (C_3_-plant dominated) was used to differentiate between new and old C. We hypothesized that (i) the proportion of added C that is stabilised as MAOC would increase with total N availability and decreasing litter C:N ratio and that (ii) this would positively affect total SOC after two years of incubation.

## Materials and methods

### Incubation experiment

To investigate the effect of litter C:N ratio on the decomposition and stabilisation of root biomass, we conducted a two-year incubation experiment with root material of different C:N ratios added to an agricultural topsoil. The soil was a long-term cropped haplic Fluvisol with a sandy loam texture (13/19/68% of clay/silt/sand) from Trier, Rhineland Palatinate (49°48′41.74″N 6°43′12.72″E) which was sampled in 2012 (Poeplau and Don 2014). A homogenised, sieved (< 2 mm) and dried (40 °C) sample of approximately 500 g from the 0–10 cm depth increment was used in the present study. The initial SOC content of the soil was 11.2 g kg^− 1^, and the total N content was 0.88 g kg^− 1^ (C:N ratio of 12.6). The root material (diameter of > 0.5 mm, but no rhizome material) was derived from a previous experiment (Poeplau et al. 2019), in which the belowground biomass of the C4 grass *Miscanthus sacchariflorus (MAXIM.)* was sampled at six different time points within a 12 months period (between April 2017 and February 2018). C and N values had previously been measured for all root samples and varied strongly with sampling depth and season, which enabled the selection and pooling of root litter samples with a gradient in C:N ratio. The newly pooled root litter samples were analysed in an elemental analyser again (LECO TruMac, St Joseph, MI, USA) and had C:N ratios of 50 (44.17 ± 0.53% C, 0.89 ± 0.01% N), 65 (43.64 ± 0.32% C, 0.67 ± 0.02% N), 85 (44.39 ± 0.07% C and 0.52 ± 0.01% N) and 124 (43.43 ± 0.44% C and 0.35 ± 0.08% N).

Prior to root litter addition and incubation, the soil water content in the mineral soil without litter was adjusted to 60% of its water holding capacity. Water holding capacity was determined by placing 5 g oven-dried, archived soil on a funnel padded with cotton wool. Water was added to the soil in excess and drained through the cotton wool. 100% water holding capacity of the soil was assumed to be reached, when no water dropped from the funnel anymore.

At the start of the incubation in October 2018, 16 g of soil, 0.4 g of root litter and 3.8 g of water were mixed thoroughly and added to 250 ml gas-tight jars. The aim was to double the C content in the soil by litter addition. This is a large and unrealistic amount of added biomass, but was chosen to ensure that after two years, the relatively weakly labelled (C4 natural abundance) root litter C would be traceable in all fractions. Before addition, the root material was milled to a particle size of approximately 2 mm to ensure homogenisation and facilitate decomposition. For each of the four different C:N ratio treatments, five replicates were incubated. Treatments will be referred to as CN50, CN65, CN85 and CN124 in the following. In addition, soil without litter material as a control (CON) as well as soil with CN124 litter material plus additional nitrogen (CN124N) were also prepared and incubated in five replicates. In the CN124N treatment, N was added as ammonium nitrate (NH_4_NO_3_) to adjust the C:N ratio of the input to 50, i.e. equal to the root litter treatment with the lowest C:N ratio. The NH_4_NO_3_ was added with the water, so completely dissolved. A total of 30 jars were incubated (6 treatments x 5 replicates) at 20 °C in the dark. Gas-tight jars were opened every two to three weeks for several minutes to allow a full exchange of the headspace with the room atmosphere. This interval was considered sufficient due to the wide volumetric headspace to soil ratio of ~ 25. Total weight of each jar was noted at the beginning of the experiment to readjust water contents if necessary. However, all bottles were gas tight and no significant change in weight was detected within the first three months, so the weighing of the jars was stopped thereafter. Whenever the jars were opened, the soil was gently shaken and stirred to stimulate microbial activity and thus maximise overall SOC decomposition.

Soils were sampled after six, 12 and 24 months to follow the fate of added root C into different SOC pools as well as its effect on native SOC: Each time, approximately one third of the initial soil sample was taken out from each jar (7–8 g) to acquire equal amounts of aliquot from each sampling date and treatment. All aliquot samples were oven dried at 40 °C until weight constancy.

### Fractionation and analysis of soil samples

During photosynthesis, C_3_ plants discriminate the stable C isotope ^13^ C stronger than C_4_ plants, such that C4 plant tissue has a greater δ^13^C signature than C_3_ tissue. This difference in the natural abundance of δ ^13^ C between the C_4_ plant *Miscanthus* and a soil with SOC predominantly from C_3_ sources can be used to trace the added C and follow its fate in the soil (Balesdent et al. 1987). In this study, the abundance of ^13^ C, expressed as relative abundance (δ^13^C) compared to the international Vienna Pee Dee Belemnite standard, was measured using an isotope ratio mass spectrometer (DeltaPlus, Thermo Fisher Scientific, Waltham, MA, USA) coupled to an elemental analyser (CE Instruments FLASH EA 1122 NA 1500, Wigan, UK). The average δ^13^C value of the *Miscanthus* root material was − 14.2 ± 0.2‰, which no detectable correlation between δ^13^C and litter C:N ratio, the average initial δ^13^C value of the soil was − 26.2 ± 0.2‰.

Around 0.5 g of each aliquot sample taken at each sampling date was milled for δ^13^C and total SOC determination. The rest of the aliquot sample was used to conduct a particle size fractionation to distinguish between mineral-associated organic carbon (MAOC) and particulate organic carbon (POC) (Lavallee et al. 2020). This was done, slightly modified, according to the protocol described in Just et al. (2021). In brief, 4–6 g of sample was added to 150 ml deionized water in a 250 ml glass beaker and subjected to ultrasonication (100 J ml^− 1^) with a 13 mm sonotrode at an immersion depth of 1.5 cm. According to Just et al. (2021), 100 J ml^− 1^, can disperse soils with moderately high clay contents equally well as the more commonly used 450 J ml^− 1^ but avoids overheating of the sample solution, which could result in the loss of OC. After dispersion, the suspended soil was wet-sieved over a 20 μm sieve placed on a 1-liter glass beaker. An aerosol pump spray was used to flush the fine particles through the sieve and 800–1000 ml water was enough to completely separate coarse and fine fractions, which was assumed to be achieved when the rinsing water was clear. The coarse fraction (> 20 μm) was immediately retrieved and dried at 40 °C. The suspended fine fractions were centrifuged for 15 min at 2600 rpm. To ensure a complete recovery of the fine fraction (< 20 μm), we added 0.2 g of CaCl_2_ as a flocculation agent. After centrifugation, the supernatant was discarded and the fine fraction was transferred into an evaporating dish and dried at 40 °C. All fractions were weighed, and milled. Average mass recovery was 98.2 ± 0.7%. According to Lavallee et al. (2020), the SOC within the fine and coarse soil fractions will be referred to as MAOC and POC in the following, representing stabilised and rather labile SOC, respectively. Organic C contents as well as the δ^13^C value of all fractions was determined as described above. To calculate the fraction of new C_4_-derived C (${f}_{C4}$) in the bulk soil and fractions at time *i*, the following mixing-equation was applied (Balesdent et al. 1987):

$${f}_{C4}=\frac{{\delta }^{13}C \left({soil/fraction}_{i}\right)-{\delta }^{13}C \left(reference \ soil/fraction\right)}{{\delta }^{13}C \left(C4 litter\right)-{\delta }^{13}C \left(reference \ soil/fraction\right)}$$

Thereby, the δ^13^C value of the reference soil or fraction was that of the CON treatment at the start of the experiment, since no clear trend in ^13^ C abundance was found for the CON treatment over the course of the experiment. The δ^13^C value of the POC fraction in the CON treatment was on average approximately 0.5‰ more negative than the MAOC fraction of the CON treatment and thus corresponding δ^13^C values were used for each fraction. A total of four different SOC pools were calculated, by using constant mass proportions of coarse and fine soil: POC_old_ and POC_new_, as well as MAOC_old_ and MAOC_new_, whereby new and old refers to *Miscanthus* C_4_ litter-derived and native C of the C_3_-plant dominated soil, respectively. Furthermore, POC_total_ and MAOC_total_ were calculated as sums of new and old C in each fraction and the same was done for bulk SOC. In this way, average C recovery (sum of all four pools divided by bulk SOC) was 102 ± 13%. The amount of mineralised root-derived N was estimated by a mass balance approach using the loss of C_4_-derived C and the C:N ratio of the respective root litter.

Due to the fact that the root litter material was obtained at different phenological stages of the perennial *Miscanthus* plant and different soil depths, we considered that the material would also differ in other properties. To assess root litter composition and quality, we applied mid-infrared spectra analysis by using diffuse reflectance infrared Fourier transform (DRIFT) spectroscopy (TENSOR 27, spectrophotometer, Bruker, Fällanden, Switzerland). Four replicates of each individual root litter were analysed in the range of 4000 − 400 cm^− 1^ with a resolution of 4 cm^− 1^ and an average of 64 scans per replicate. All samples were milled, dried at 40 °C and stored in a desiccator prior to the analysis. Background correction was performed with KBr and CO_2_ and H_2_O interferences were corrected using OPUS (version 8.2) internal correction. Maximum peak absorbance was used to compute compound ratios and assess relative differences in aliphatic to aromatic (Aliphatic:Aromatic) and polysaccharides- and lignin-like (Polysaccharides:Lingin) compounds. To consider shifts in N containing compounds, we used absorbance peaks diagnostic for amide I, amide II and amide III bonds and computed ratios relative to the aliphatic bonds (Aliphatic:Amide I, Aliphatic:Amide II, Aliphatic:Amide III). All selected band ranges for the compounds are given in Table 1.

**Table 1:** Compounds and bond of vibration with selected range of wave number bands for the maximum absorbance determination

	Compound (vibrating bonds)	Range of band	Reference
1	Aliphatic (C-H)	2990 − 2915 cm^− 1^	(Demyan et al. 2012; Laub et al. 2020)
2	Aromatic (C = C and COO^−^)	1660 − 1600 cm^− 1^	(Demyan et al. 2012; Laub et al. 2020)
3	Amide I (C = N and C = O)	1658 − 1652 cm^− 1^	(Ji et al. 2020)
4	Amide II (N-H and C-N)	1548 − 1540 cm^− 1^	(Ji et al. 2020; Peltre et al. 2014)
5	Lignin (C = C)	1512 − 1504 cm^− 1^	(Peltre et al. 2014; Spaccini and Piccolo 2007)
6	Amide III (C-N, C-H, C-C)	1320 − 1230 cm^− 1^	(Ji et al. 2020; Peltre et al. 2014)
7	Polysaccharides (C-O)	1180 − 1140 cm^− 1^	(Peltre et al. 2014; Spaccini and Piccolo 2007)

### Statistical analysis

Linear regression models were used to evaluate the effect of litter C:N ratio on SOC dynamics, including total, new and old bulk SOC, as well as total, new and old MAOC and POC at each point in time. Significance was assessed at *p* < 0.05 and fits are only shown when a significant correlation was detected. Additionally, one-way analysis of variance (ANOVA) with the Tukey’s HSD post-hoc test was used to test for differences in SOC contents between treatments after 24 months (reported in Table 1). Model residues were checked for approximate normal distribution using QQ-plots, which was given in all cases. All statistics and plots were done in R version 4.2.1 (R Core Team 2020) using the *ggplot2* (Wickham 2016), *ggpubr* (Kassambara 2020) and *agricolae* (de Mendiburu 2019) packages.

## Results

**Fig. 1:**
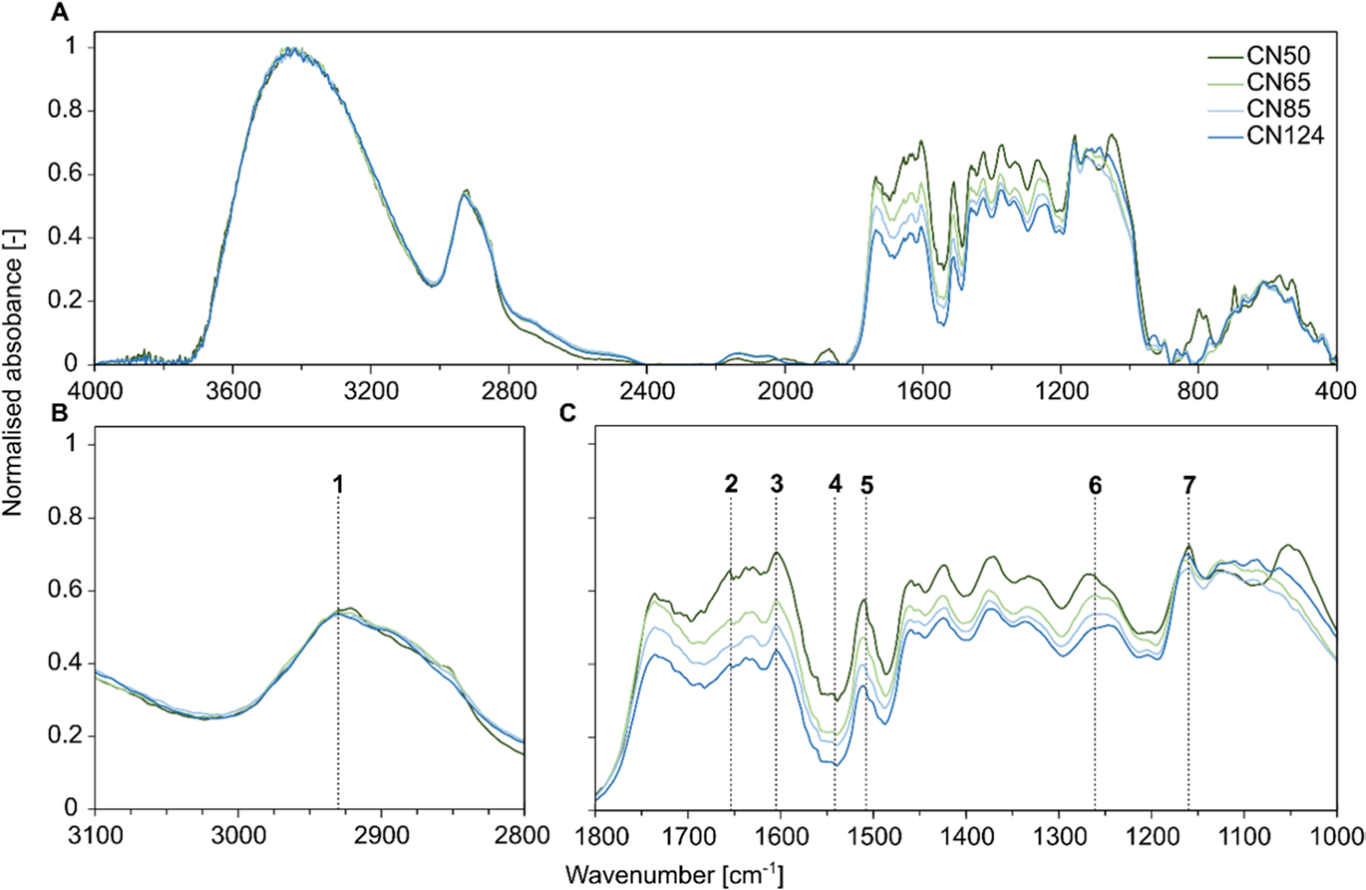
Mid-infrared absorbance spectra of *Miscanthus* root litter (**A**) and detailed 3100 − 2800 cm− 1 (**B**) and 1800 − 1000 cm− 1 (**C**) ranges with compounds 1–7 as described in Table 1. All spectra are normalised to its maximum absorbance for comparison

**Fig. 2:**
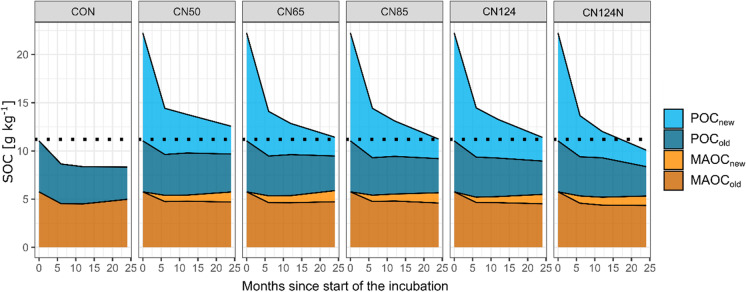
Area plot with average cumulative soil organic carbon (SOC) contents in all fractions in the course of the two-year incubation. Dotted line at 11.2 g kg^− 1^ SOC depicts the initial SOC content of the soil without litter. POC = particulate organic C, MAOC = mineral-associated organic C, new = derived from added root litter, old = native SOC

**Fig. 3:**
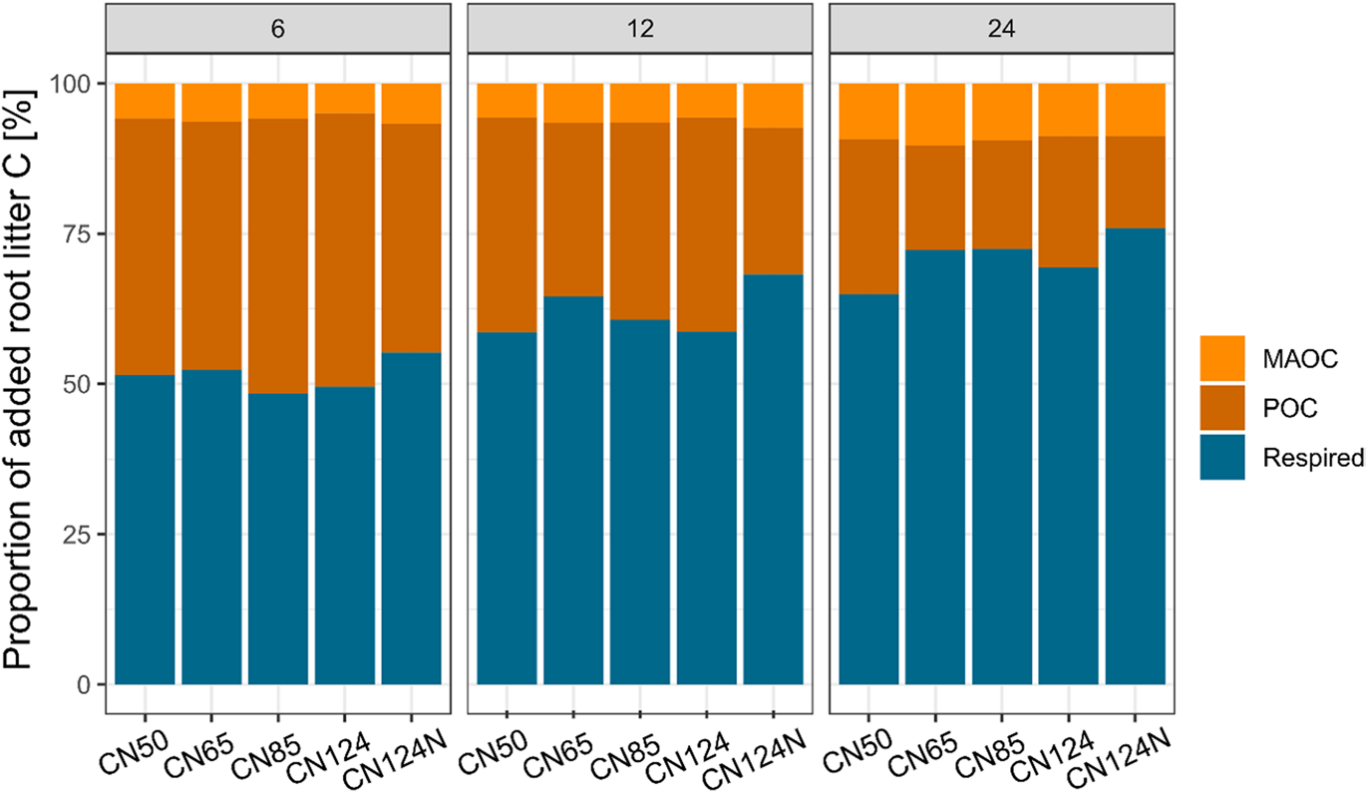
Mass balance derived proportions of added root litter carbon (C) that was recovered as mineral-associated C (MAOC), particulate organic C (POC) or lost via respiration after 6, 12 and 24 months of incubation

In the course of the two-year incubation experiment, the incubated soils lost on average 47.3 ± 3.5% of the total SOC, which initially consisted of of 50% *Miscanthus* root litter C (new) and 50% native SOC (old) (Table 2). The greatest absolute and relative losses were observed in the POC_new_ fraction (-80.3 ± 4.9%), i.e. the *Miscanthu*s root fraction (Table 2; Figs. 1 and 2). In contrast, POC_old_, which is usually treated as the relatively labile fraction with a fast turnover, was much less depleted (-33.5 ± 11.2%), indicating that these two fractions were kinetically not identical. Accordingly, the difference between the loss of POC_old_ and MAOC_old_ (-20.4 ± 3.1%) was relatively small, especially because some of the mineralised POC_old_ was most likely transformed into MAOC_old_ in the course of the two years, so that the mineralisation of the initially present MAOC_old_ was even slightly higher than the quantified change. In the case of the *Miscanthus* root litter, 9.3 ± 11.2% was transformed into MAOC_new_ (Table 2). Thus, on average 70% of the added root litter was lost as CO_2_ after two years, while 20 and 10% were recovered as POC and MAOC respectively (Fig. 3).

**Table 2:** Measured relative changes [%] in soil organic carbon (SOC), particulate organic carbon (POC) and mineral-associated organic carbon (MAOC) after two years of incubation for each of the investigated treatments

Treatment	ΔSOC	ΔPOC_new_	ΔPOC_old_	ΔMAOC_new_	ΔMAOC_old_
CN50	-42.5 ± 3.0 a	-74.2 ± 2.1 a	-25.5 ± 5.7 a	9.3 ± 0.7 a	-18.3 ± 2.3 ab
CN65	-47.7 ± 1.87 ab	-82.6 ± 3.8 bc	-32.1 ± 11.3 a	10.3 ± 1.1 a	-17.8 ± 2.6 a
CN85	-48.0 ± 3.1 b	-81.9 ± 3.9 bc	-33.1 ± 18.1 a	9.5 ± 1.2 a	-20.0 ± 1.6 ab
CN124	-48.9 ± 3.0 b	-78.1 ± 3.3 ab	-34.6 ± 4.0 a	8.7 ± 0.8 a	-21.5 ± 0.9 bc
CN124N	-49.2 ± 2.5 b	-84.7 ± 3.2 c	-42.4 ± 7.8 a	8.7 ± 0.6 a	-24.5 ± 1.8 c
Average	-47.3 ± 3.5	-80.3 ± 4.9	-33.5 ± 11.2	9.3 ± 1.0	-20.4 ± 3.1

The ΔSOC includes both, new and old carbon (C). Changes in MAOC_new_ are given as proportion of the initially added root-derived C, while changes in all other fractions are given as proportion of the initial amount of C in the respective fraction of the CON treatment. Letters indicate significant differences between treatments (*p* < 0.05)

**Fig. 4:**
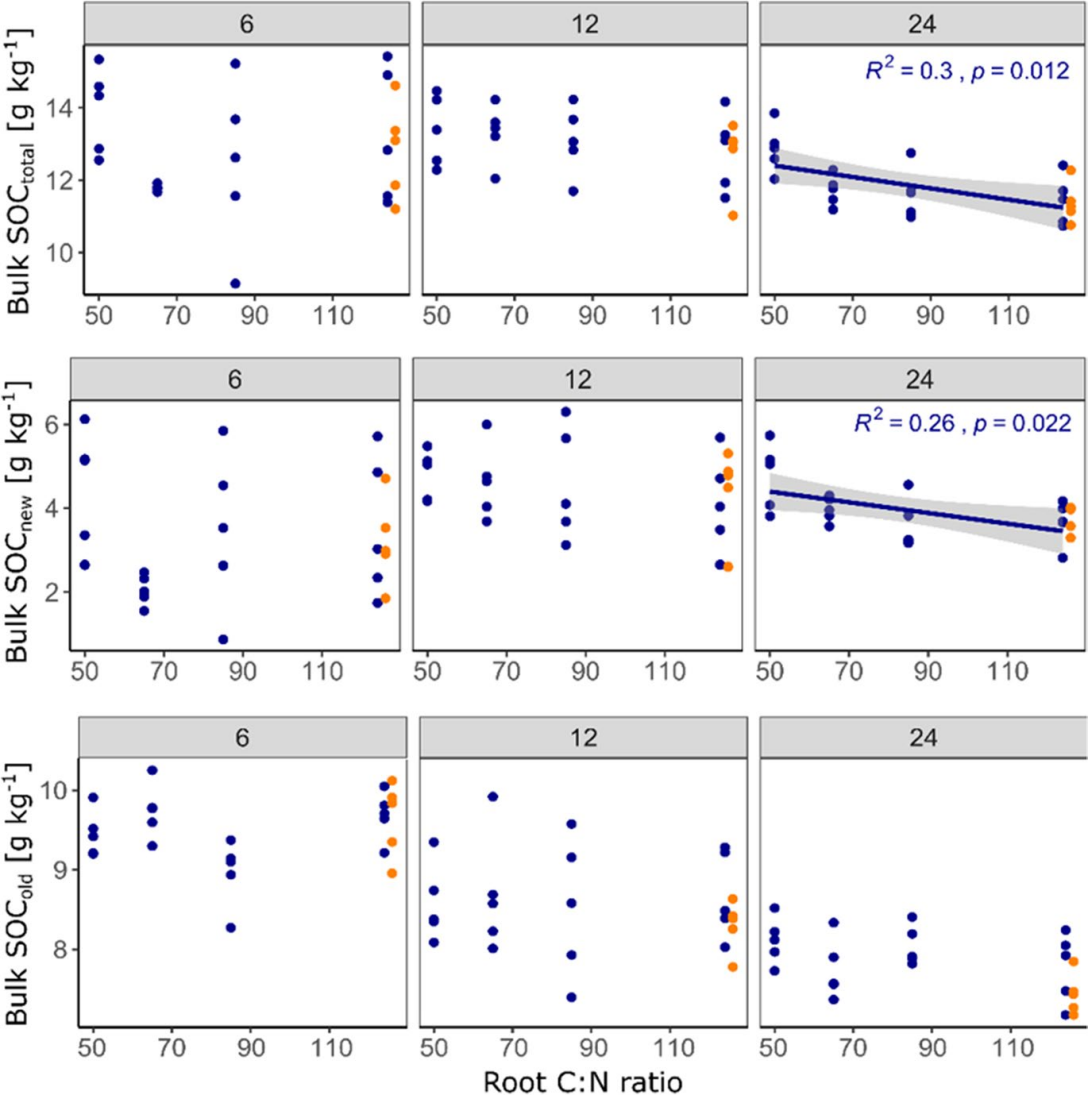
Observed soil organic carbon (SOC) contents after six, 12 and 24 months as a function of root carbon to nitrogen (C:N) ratio. Only significant correlations (*p* < 0.05) are depicted with a regression line, 95% confidence interval and summary statistics. Points in orange represent the CN124N treatment (root C:N ratio of 124 plus mineral N addition adjusted to C:N of 50), which were not included in the regression analysis

**Fig. 5:**
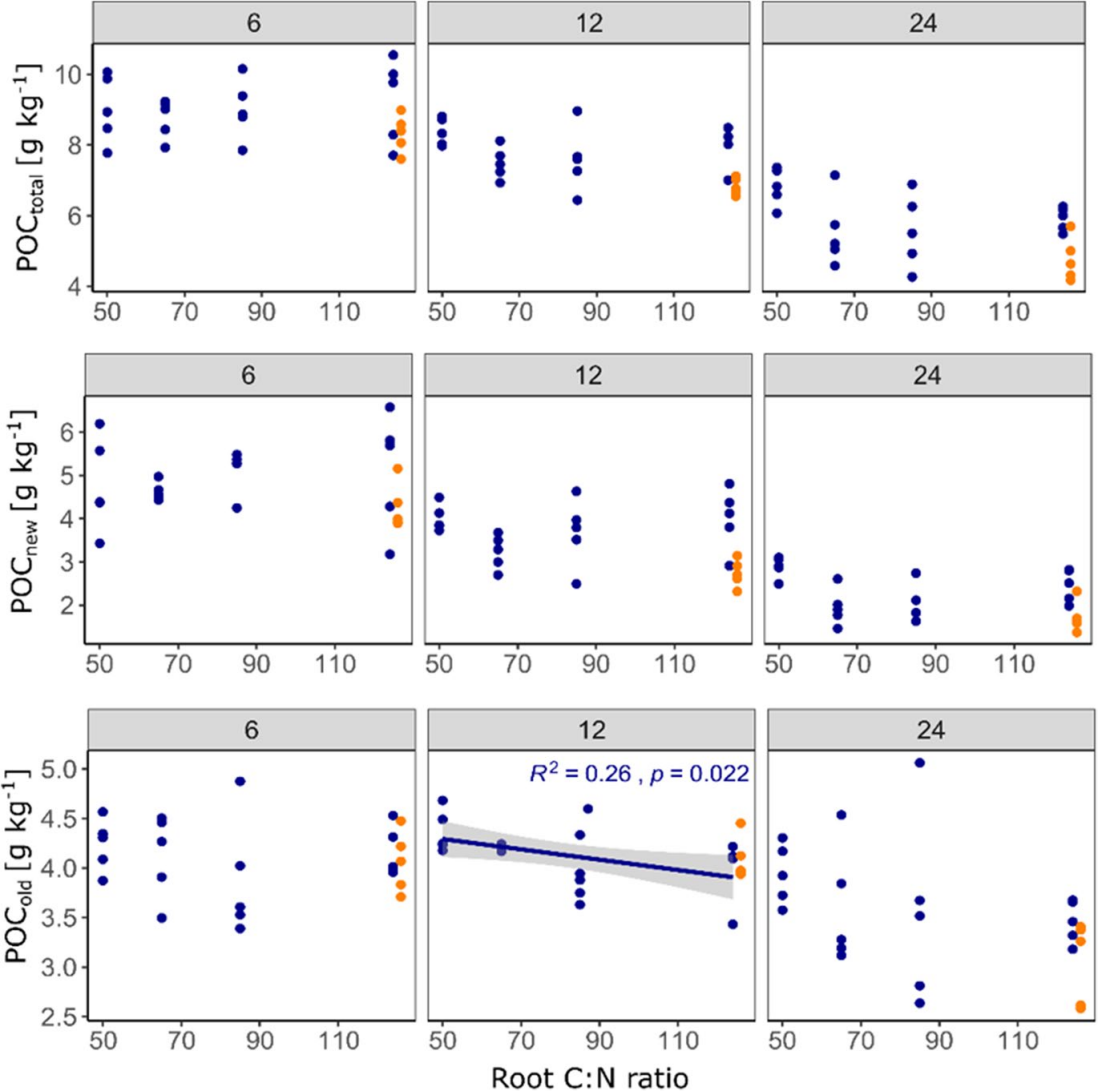
Observed particulate organic carbon (POC) contents after six, 12 and 24 months as a function of root carbon to nitrogen (C:N) ratio. Only significant correlations (*p* < 0.05) are depicted with regression line, 95% confidence interval and summary statistics. Points in orange represent the CN124N treatment (root C:N ratio of 124 plus mineral N addition adjusted to C:N of 50), which were not included in the regression analysis

**Fig. 6:**
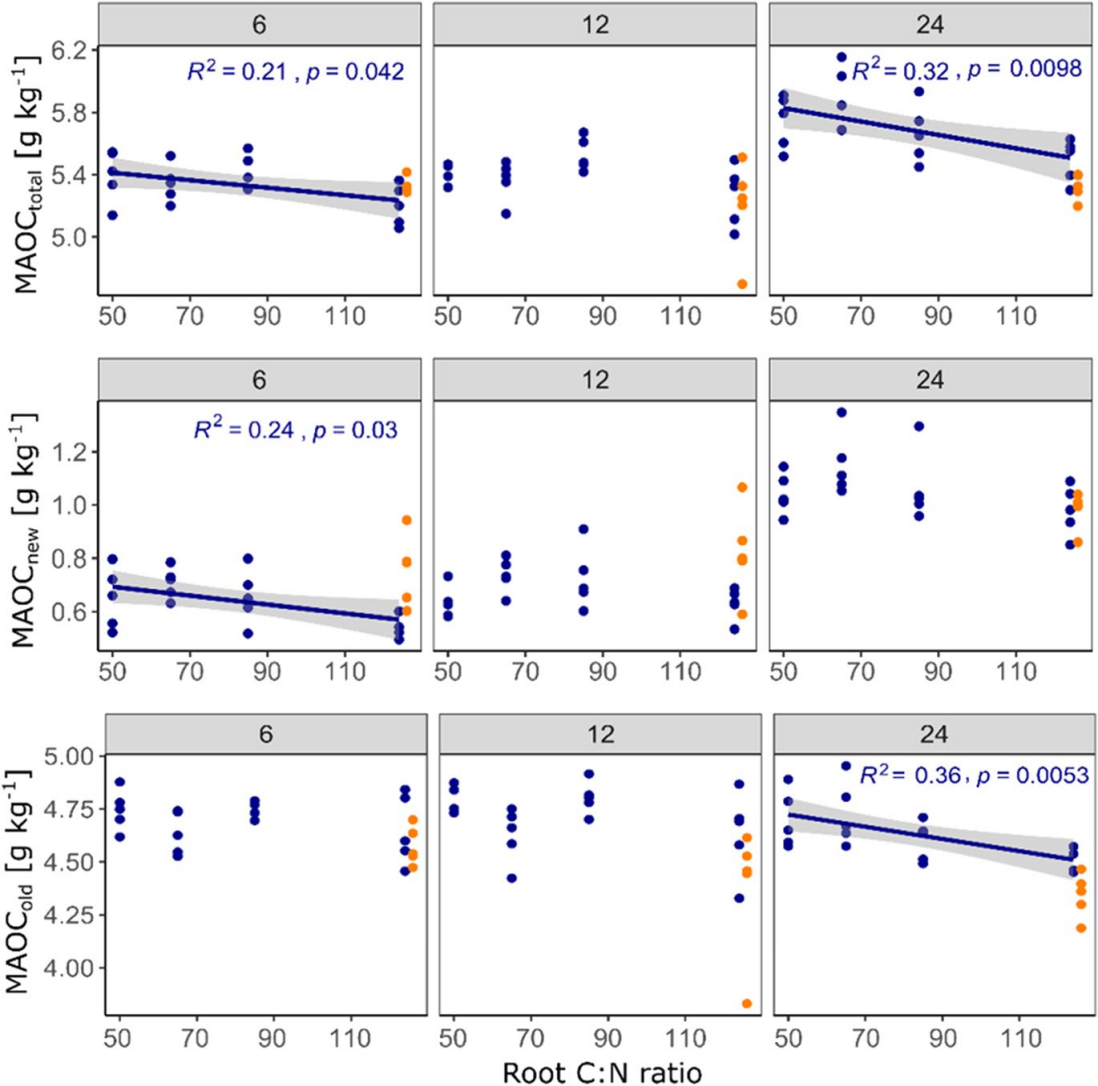
Observed mineral-associated organic carbon (MAOC) contents after six, 12 and 24 months as a function of root carbon to nitrogen (C:N) ratio. Only significant correlations (*p* < 0.05) are depicted with regression line, 95% confidence interval and summary statistics. Points in orange represent the CN124N treatment (root C:N ratio of 124 plus mineral N addition adjusted to C:N of 50), which were not included in the regression analysis**Carbon (C) content in the investigated soils after two years of incubation as a function of nitrogen (N) mineralised from the added roots (calculated by mass balance). Only significant correlations (*p* < 0.05) are depicted with regression line, 95% confidence interval and summary statistics. Points in orange represent the CN124N treatment (root C:N ratio of 124 plus mineral N addition adjusted to C:N of 50), which were not included in the regression analysis

**Fig. 7:**
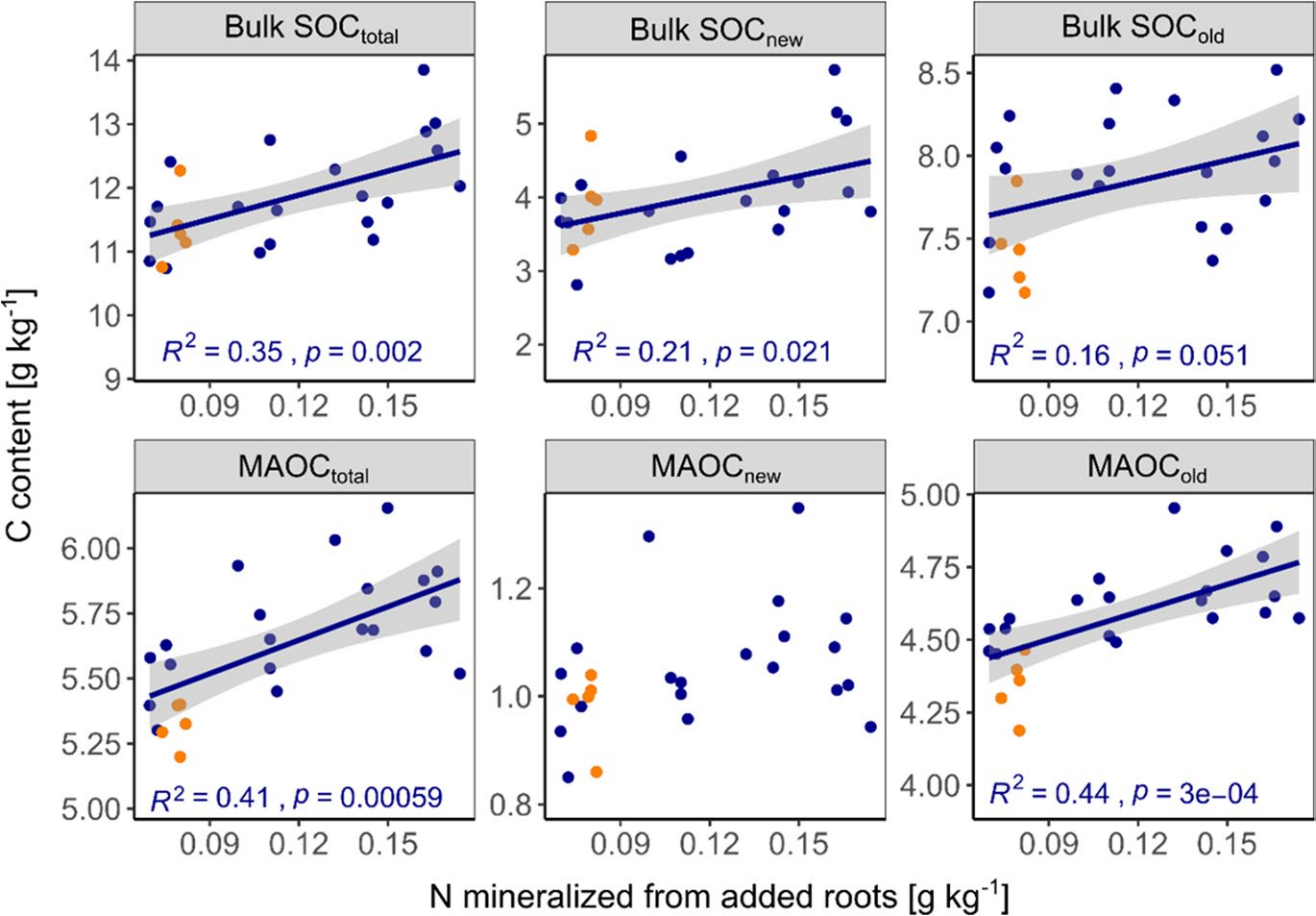
Carbon (C) content in the investigated soils after two years of incubation as a function of nitrogen (N) mineralised from the added roots (calculated by mass balance). Only significant correlations (*p* < 0.05) are depicted with regression line, 95% confidence interval and summary statistics. Points in orange represent the CN124N treatment (root C:N ratio of 124 plus mineral N addition adjusted to C:N of 50), which were not included in the regression analysis

For changes in bulk SOC and all of its compartments, including new and old POC and MAOC, we found more or less clear effects of root litter C:N ratio (Table 2): the final total bulk SOC content was significantly negatively correlated with litter C:N ratio (Fig. 4), which was strongly driven by the negative correlation of C:N ratio and new bulk SOC (Fig. 4). For new and old POC, there was a strong negative tendency (Table 2), yet significance was hardly detected due to large scatter in the data (Fig. 5). The only significant negative correlation of POC_old_ and C:N ratio of added litter was found in the samples taken after 12 months. Finally, MAOC_total_ was, after 24 months of incubation, also negatively affected by the litter C:N ratio, which was strongly driven by the dynamic of MAOC_old_ for which we found the strongest negative correlation with litter C:N ratio among all investigated C pools (p = 0.0052, Fig. 6). In contrast, MAOC_new_, i.e. the formation of MAOC from added root litter was negatively affected by C:N ratio after 6 months, but not significantly so after 24 months. Figures 4, 5 and 6 also highlight that significant correlations were mostly found after two years of incubation only. Figure 7 indicates that the final amount of C in bulk SOC and MAOC was significantly and positively correlated with the amount of mineralised root biomass N, as estimated from the loss of litter-derived C. Also in this case, the strongest correlation was found for MAOC_old_.

**Fig. 8:**
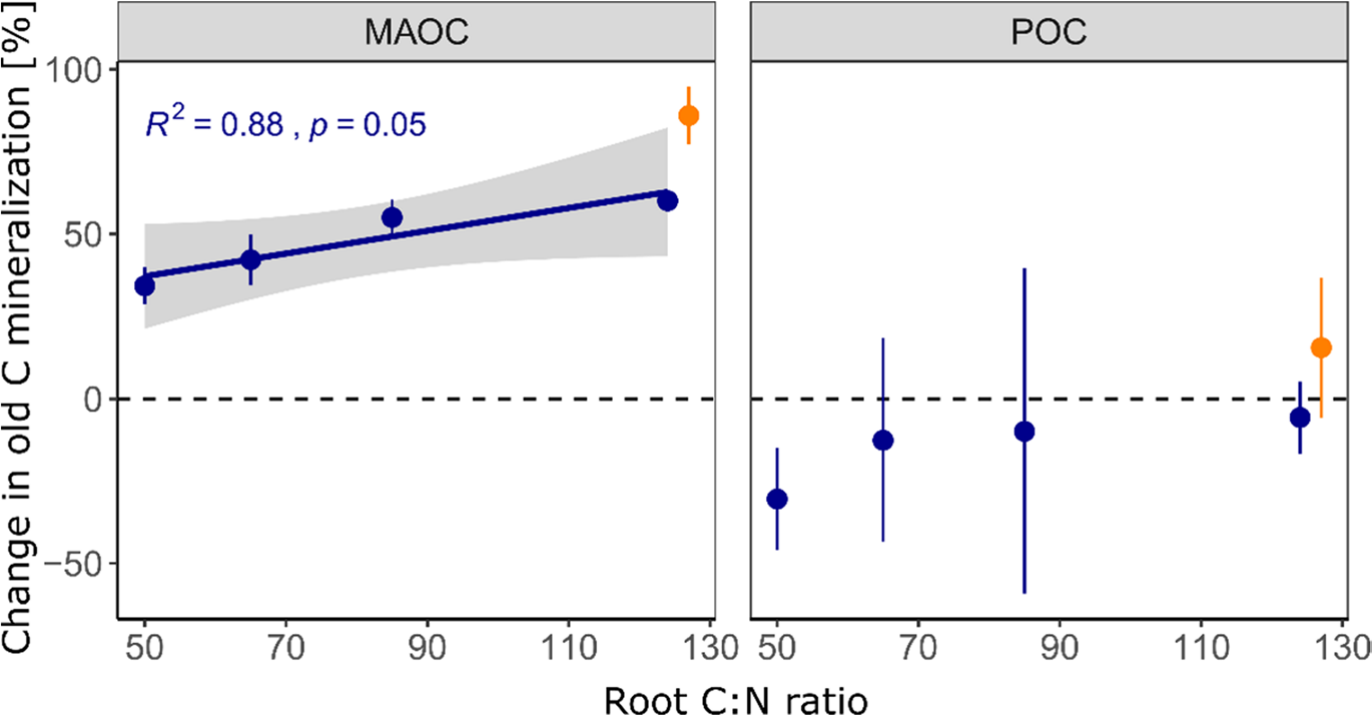
Change in old carbon (C) mineralisation upon litter addition after two years as a function of root litter C:N ratio for mineral-associated organic C (MAOC) and particulate organic C (POC). Depicted values represent mean relative differences in C loss between the root-litter amended soils (*n* = 5) and the average C loss in the unamended reference soil (*n* = 1) with standard deviation. Only significant correlations (*p* < 0.05) are depicted with regression line, 95% confidence interval and summary statistics. Points in orange represent the CN124N treatment (root C:N ratio of 124 plus mineral N addition), which were not included in the regression analysis

A comparison with the incubated reference soil (CON) revealed that after two years of incubation, significantly more MAOC_old_ was lost from all soils amended with litter (Fig. 8). A positive correlation with C:N ratio was found, i.e. the MAOC_old_ decay was stimulated the most, when added root N was lowest. In contrast, loss of POC_old_ tended to be lower in the root litter amended soils as compared to the reference soil and the correlation with C:N ratio was not significant due to highly variable observations.

A further treatment was CN124N, in which the added litter had a C:N ratio of 124, but mineral N was added to adjust the total amount of added N to an amendment C:N ratio of 50. Despite the equal total N addition, C dynamics were not at all comparable to the CN50 treatment. Instead, this treatment tended to have the highest losses of total SOC, POC_new_, POC_old_ and MAOC_old_, as well as among the lowest formation of MAOC_new_ (Table 2; Figs. 2, 3, 4, 5, 6 and 7). It was thus much closer to the CN124 treatment than the CN50 treatment. This might indicate that N availability was not the only driver of the observed C dynamics after adding litter from the same plant with various qualities.

Results from the DRIFT analysis revealed that the litter material differed in its relative composition (Table 3). With increasing C:N ratio, the root litter was relatively enriched in aromatic and polysaccharide-like compounds as compared to aliphatic and lignin-like compounds. In general, the largest shifts in the spectral absorbance were at those wave numbers diagnostic for the aromatic and amide compounds, with decreasing intensity at higher C:N ratios (Fig. 1). This is indicating that N was preferentially stored in more lignified roots. The Aliphatic:Aromatic and Polysaccharide:Lignin ratios were highly correlated with litter C:N ratio (Table 3). Accordingly, the Aliphatic:Amide ratios increased with increasing C:N ratios. Total and new bulk SOC after two years of incubation were similarly well or slightly better predicted by the chosen DRIFT indicators than by litter C:N ratio, while POC was generally not correlated with any of the litter quality indicators (Table 4). All indicators were significantly correlated with the content of MAOC_old_ after two years, which highlights the strong effect of litter quality on MAOC_old_ dynamic.

**Table 3:** Compound ratios as derived from DRIFT measurements and maximum peak absorbance for each of the four root litter treatments (CN50-CN124) with correlation coefficient (R²) of the relationships between litter carbon to nitrogen (C:N) and compound ratio

Compound ratio	CN50	CN65	CN85	CN124	R^2^
Aliphatic:Aromatic	0.78	0.94	1.06	1.23	0.97
Polysaccharide:Lignin	1.26	1.44	1.66	2.06	0.99
Aliphatic:Amide I	0.84	1.04	1.19	1.34	0.93
Aliphatic:Amide II	1.73	2.52	2.86	4.06	0.97
Aliphatic:Amide III	0.86	0.92	1.00	1.06	0.95

All spectra and compounds specific absorbance bands are given in Table 1; Fig. 1

**Table 4:** Correlation coefficients [R²] for each root quality indicator (C:N ratio or DRIFT compound ratios) and the observed organic carbon contents [g kg^− 1^] in the different C pools investigated after two years of incubation; ns = non-significant (*p* > 0.05)

Quality indicator	Bulk SOC			POC			MAOC		
	Total	New	Old	Total	New	Old	Total	New	Old
C:N ratio	0.31	0.27	ns	ns	ns	ns	0.31	ns	0.36
Aliphatic:Aromatic	0.36	0.34	ns	ns	ns	ns	0.25	ns	0.32
Polysaccharide:Lignin	0.31	0.27	ns	ns	ns	ns	0.31	ns	0.36
Aliphatic:Amide I	0.41	0.37	ns	ns	ns	ns	0.23	ns	0.31
Aliphatic:Amide II	0.36	0.29	ns	ns	ns	ns	0.25	ns	0.31
Aliphatic:Amide III	0.41	0.31	ns	ns	ns	ns	0.27	ns	0.34

## Discussion

### Overall dynamic of organic carbon in bulk soil and fractions

The two-year incubation experiment revealed several interesting findings regarding the overall dynamic of SOC. First of all, we expectedly observed a rapid depletion of the freshly added labile C. Under close to optimal abiotic conditions, approximately half of it was respired within 6 months (Fig. 2). After two years, 20% of the initially added root litter C was left as POC, another 10% became MAOC, thus 70% was respired (as a result of mass balance). This underlines the fast turnover of freshly added plant material in agricultural soils under optimum conditions, even without soil fauna. It is also well in line with field observations, that only a very small fraction of fresh C inputs adds to the refractory C pool (Berthelin et al. 2022; Poeplau et al. 2021). At the same time, the old C_3_-derived POC in the soil was not decomposed at a similar pace as the freshly added material. After two years, only 33% were lost from the initial POC_old_. This fits well to the exponential decay of litter and the established view that a certain fraction of POC or added litter has a much slower turnover than the labile compounds that are lost at the very beginning (Keuskamp et al. 2013; Prescott 2005). In fact, on average POC_old_ was mineralised only 67% faster than MAOC_old_, which raises the question if the most recent concept of kinetic C pools in soils, i.e. POC and MAOC as fast (years to decades) and slow (decades to centuries) cycling pools resembles an oversimplification (Lavallee et al. 2020). Surely, on average the operationally defined and isolated POC and MAOC are functionally and kinetically contrasting C pools. However, this study and also others revealed a certain functional overlap: a considerable proportion of MAOC must be relatively fast cycling, while a certain proportion of POC is slow cycling (Poeplau et al. 2018a; Ridgeway et al. 2022; Torn et al. 2013).

Furthermore, sampling the soils after 6, 12 and 24 months revealed that a longer incubation time increased the likelihood of finding significant treatment effects. Results became clearer and, in most cases, the variability across replicates decreased (Figs. 4, 5 and 6). The formation of MAOC is a slow and steady process, while the mineralisation kinetics of freshly added C follows a more exponential pattern (Fig. 2). Following the temporal dynamics, if possible over years, is thus necessary to develop in-depth understanding of the fate of newly added C in soils and potential responses of native SOC (Lecerf et al. 2011; Prescott 2005).

### The effect of litter quality on mineralisation and stabilisation of C

The microbial efficiency matrix stabilisation (MEMS) framework assumes that high quality litter such as labile substrates rich in nutrients and water-soluble compounds will be stabilised in the soil to a higher proportion than chemically recalcitrant compounds (Cotrufo et al. 2013). This is mainly explained by shifts in microbial C use efficiency (CUE), leading to a higher production of microbial biomass and necromass that can become stabilised in the soil by mineral interaction (Nicolardot et al. 2001; Sokol et al. 2019). When the substrate C:N ratio becomes higher and microbial growth becomes N limited, microbes have to mine for N and thus respire a relatively high amount of C to meet their stoichiometric and energetic needs (Craine et al. 2007; Manzoni et al. 2017; Spohn and Chodak 2015). Furthermore, depolymerisation of protected polymers by extracellular enzymes is also costly and reduces microbial CUE (Mganga et al. 2022). In this study microbial growth was certainly N limited, since the C content in the soil was doubled, while depending on the litter treatment only little N was added. Indeed, after two years of incubation, the C:N 124 treatment had the lowest total bulk SOC contents, while the C:N 50 treatment had the highest. This indicates that litter C:N ratio indeed had a strong effect on total SOC dynamics in the studied soil, which is in line with the results of Nicolardot et al. (2001): the authors incubated 48 different crop residues for a total of six months and detected that the so called “humification coefficient” (indicator for the efficiency of SOC formation) decreased with increasing C:N ratio. In our study, the two SOC fractions and their new (*Miscanthus*-derived) and old (native) parts revealed the same tendencies, i.e., less of the individual fraction was recovered after two years with increasing C:N ratio (Figs. 4 and 5). However, the fluxes most affected by litter quality were not the losses of new litter C, nor the formation of new MAOC. Instead, the mineralisation of old, N-rich mineral-associated organic matter was most strongly modified by litter C:N ratio. Thus, a lower N content in the litter caused a higher mineralization, remarkably of the old MAOC fraction (Figs. 6 and 7). This did not fully agree with our hypothesis that the formation of new MAOC increases with decreasing litter C:N ratios, but is in line with the N mining theory (Craine et al. 2007). In fact, litter quality has been found to be a major driver of the priming effect also in other studies (Mo et al. 2022; Fanin et al. 2020). This trend became clearer over time, indicating that the observed “priming for mining” cannot be classified as a short-term mechanism in this case, which is often included in the definition of the priming effect as such (Kuzyakov et al. 2000). Interestingly, positive priming was only observed for MAOC, not for POC. This might underline the findings of Murphy et al. (2015), who estimated a C:N ratio of 5 in the primed organic matter in comparison to 20 in the decomposed organic matter of the unamended control soil. The authors hypothesized that priming might indeed be a distinct and active microbial process to gain energy in search for specific, nutrient-rich compounds. This would further be in accordance with Chen et al. (2014), who observed increased activity of certain enzymes during priming. It suggests that the stability of native MAOC can, among other factors, be described as a function of C input quality.

The fact that native POC mineralisation was not increased, but rather decreased with litter addition (negative priming), is in contrast to recent findings of Su et al. (2023), who found less positive priming of old and stable C as compared to young, more labile C. However, we believe that (i) also in our study it was most likely not the oldest and most protected MAOC that was primed and (ii) the large amount of freshly added litter (new POC) might have been a sufficient and certainly more accessible source of energy so that old POC mineralisation was actually decreased. Finally, MAOC is acknowledged to have higher nutrient contents (e.g. N) and, once destabilised, also lower activation energy than POC (Kleber et al. 2015).

### Effect of mineral nitrogen addition on mineralisation and stabilisation of carbon

Ammonium nitrate addition did not have the expected effects of alleviating microbial N limitation. Kirkby et al. (2014) demonstrated that straw amended in combination with N, phosphorus (P) and sulphur (S) led to a much higher formation of MAOC than straw without nutrients in a 56-days incubation experiment. At the same time, the authors found less priming of native SOC when straw was added together with nutrients, at least in two out of four soils. Their findings are in line with the litter C:N ratio results of the present study as discussed in the previous section. However, they are very much in contrast to the observation in this study for the N-amended treatment: despite the fact that mineral N was used to adjust the C:N ratio of the litter with the highest C:N ratio (124) to the one with the lowest C:N ratio (50), this treatment did not yield similar results as the litter with a C:N ratio of 50. Instead, after two years of incubation it tended to be the most C-depleted treatment of all. This was true for total SOC, but also for all investigated fractions. It was therefore more comparable to the litter with a C:N ratio of 124 than to that with a C:N ratio of 50. Also, the priming effect in the course of the two years was strongest in the N amended treatment (CN124N), for both MAOC and POC. The more positive priming effect upon labile C plus N addition in comparison to only labile C addition was observed before (Chen et al. 2014; Moran et al. 2005). Such a response is described in the “stoichiometric decomposition theory”, which suggests that microbial activity and organic matter decomposition is highest when C and N inputs better match microbial demands (Chen et al. 2014; Hobbie 2000). This apparent opposite response to N addition as compared to the N mining theory has been explained by a shift in the functional composition of the microbial community: rapidly growing r-strategists may profit most from substrate and nutrient additions and lead to a strong increase in the microbial community and enzyme activity in general (Chen et al. 2014). The authors also argue, that although both theories seemingly contradict each other at the first glance, they might co-occur spatially and temporally, or follow each other. Indeed, also in this study we detected a certain temporal trend in the relative responses of the N amended treatment. This is especially true for the formation of new MAOC, which was highest in the N amended treatment after 6 and 12 months of incubation. That observation could suggest that there was indeed the highest microbial biosynthesis and entombing of microbial residues in the N amended treatment during the first year, which would be in line with the short-term incubation study of Kirkby et al. (2014). However, it tended to be lowest after 24 months (Fig. 6). Potentially, the strong difference in the temporal pattern of MAOC formation between CN124N and CN50, was related to actual N availability at the beginning of the experiment: In the CN50 treatment, the added N was fully bound in organic matter, while it was readily available in the CN124N treatment. These contrasting starting conditions might have triggered various mechanisms in opposing directions, including gaseous losses of the added N. Again, this highlights the importance of studying temporal dynamics for the overall understanding of the fate of litter-derived C. Care should be taken in the extrapolation of observed short-term responses.

Moreover, Feng and Zhu (2021) suggested that the effect of N addition on microbial metabolism and C cycling is not primarily regulated via stoichiometric needs of the microbial community, but that N addition can lead to a weakening of organo-mineral associations. In their study, after six years of N addition, the authors found a shift in the fungal community towards a higher proportion of *Sordariomycetes* and *Leotiomycetes*, both of which are known as effective producers of oxalic acid. Indeed, Li et al. (2021) found that the amount of oxalic acid was significantly increased with N addition and this acid is acknowledged to destabilise C via ligand weathering and exchange. At first glance, the observation that N addition stimulated C mineralisation in the present study might be a contradiction to the often-reported positive effect of N fertilisation on SOC stocks (Alvarez 2005; Kätterer et al. 2012; Tang et al. n.d.). However, as discussed above, the effects of N fertilisation on the soil C balance, considering inputs and outputs, are manifold. A major difference between the present incubation experiment and long-term N fertilisation is, that in long-term field N fertilisation experiments (i) net primary production is stimulated resulting in an increase in C inputs (Kätterer et al. 2012), and (ii) the C:N ratio of the residues is also affected by N-fertilisation (Poeplau et al. 2018b). A single dose of N addition to a low-N litter amendment is thus an unrealistic scenario, but showed that it does not induce a similar C dynamic as a litter amendment with a lower C:N ratio. Inorganic N addition should thus not be used to mimic changes in litter C:N ratio.

Finally, the N addition treatment might also reveal that the difference between treatments is not caused by C:N ratio alterations alone. Indeed, the *Miscanthus* root material also differed strongly in compound composition, which is acknowledged to affect its fate in the soil (Rahman et al. 2013). The abundance of lignin-like and aromatic compounds were found to relatively decrease with increasing litter C:N ratio compared to more aliphatic and polysaccharides-like compounds, which was in contrast to other studies (Pei et al. 2019; Taylor et al. 1989). However, in most studies evaluating how litter quality affects its decomposability, the litter material was from different plant species or different plant tissues. Here, we used root material from the same species sampled at different phenological stages and rooting depths. It cannot clearly be reconstructed which *Miscanthus* roots (e.g. which exact size and age class) contributed to which pool sample, but the relatively higher N content in more lignified roots might indicate that those roots were more important as nutrient storage organs. *Miscanthus* is known for high nutrient efficiency with N translocation and remobilisation being important plant internal mechanisms to avoid N losses (Leroy et al. 2022). Of course, belowground nutrient storage organs (rhizome material was excluded), need to be better protected from microbial decay than the finest roots that are mainly used for resource acquisition. As expected, amides as N-containing compounds were relatively enriched in roots with lower C:N ratio. Their accumulation can be a response to environmental stress (Rare 1990) and some amides have shown antifungal activity (Fattorusso et al. 1999). Overall, the very high correlation of the compound ratios obtained by DRIFT and the root C:N ratios in this study hampers a clear separation of N-related mechanisms from effects of molecular composition and thus chemical recalcitrance. At the same time, it also highlights that the C:N ratio is a strong indicator of substrate quality, which does not only influence the fate of litter itself, but might even more strongly control the mineralisation of native SOC.

## Conclusions

The two-year long incubation experiment conducted in this study confirmed that root litter quality, including C:N ratio and also molecular composition, can have a strong impact on overall SOC dynamics. It has been suggested in earlier works, that this is mainly related to the interplay of litter decomposability and new MAOC formation. Here, we show that litter decomposability, new MAOC formation, but also native POC and MAOC mineralisation all tended to be affected by litter C:N ratio. Thereby, the stimulation of native MAOC mineralisation as an active N-mining mechanism might be the most important mechanism to differentiate between different types of litter when describing their effect on SOC dynamics. The coupling of added litter and native SOC mineralisation deserves further attention also in SOC modelling for a more accurate and mechanistic parametrisation of the wide range of organic matter inputs to soils. At the same time, adding mineral N to compensate for N limitation and potentially alleviate microbial N-mining did not have the anticipated effect, which indicates that it is certainly too simple to explain the effect that litter quality demonstrated on SOC dynamic by stoichiometric imbalances alone.

## Data Availability

The dataset generated during the present study is available at 10.5281/zenodo.7848172.
